# Tilapia-Head Chondroitin Sulfate Protects against Nonalcoholic Fatty Liver Disease via Modulating the Gut–Liver Axis in High-Fat-Diet-Fed C57BL/6 Mice

**DOI:** 10.3390/foods11070922

**Published:** 2022-03-23

**Authors:** Hui Yu, Xiangzhou Yi, Xia Gao, Jun Ji, Zhongyuan Liu, Guanghua Xia, Chuan Li, Xueying Zhang, Xuanri Shen

**Affiliations:** 1 Hainan Engineering Research Center of Aquatic Resources Efficient Utilization in South China Sea, Hainan University, Haikou 570228, China; yuhui19970607@163.com (H.Y.); xiangzhouyi1995@hainanu.edu.cn (X.Y.); gaoxia20202021@163.com (X.G.); jijunbest@126.com (J.J.); liuzhongyuan999@126.com (Z.L.); xiaguanghua@vip.163.com (G.X.); lichuanbest@126.com (C.L.); 994257@hainanu.edu.cn (X.Z.); 2 College of Food Science and Technology, Hainan University, Haikou 570228, China; 3 Collaborative Innovation Center of Marine Food Deep Processing, Dalian Polytechnic University, Dalian 116000, China

**Keywords:** tilapia head, chondroitin sulfate, nonalcoholic fatty liver disease, gut–liver axis

## Abstract

We isolated and characterized tilapia-head chondroitin sulfate (TH-CS) and explored its biological activity and mechanisms of action as an oral supplement for nonalcoholic fatty liver disease (NAFLD) induced by a high-fat diet (HFD) in mice. The results showed that treatment with TH-CS for 8 weeks alleviated the development of NAFLD, as evidenced by the notable improvement in liver damage, blood lipid accumulation and insulin resistance (IR). Meanwhile, TH-CS treatment reduced the expression of proinflammatory cytokines and normalized oxidative stress. Additionally, the analysis of 16S rDNA sequencing revealed that TH-CS could restore gut microbiota balance and increase the relative abundance of short-chain fatty acid (SCFA)-producing bacteria. Furthermore, SCFAs produced by related bacteria can further improve lipid metabolism and IR by regulating lipid synthesis signals. In conclusion, TH-CS is an effective dietary supplement for the prevention of NAFLD, and may serve as a potential supplementary treatment for lipid-related metabolic syndrome.

## 1. Introduction

Nonalcoholic fatty liver disease (NAFLD) is a clinicopathological syndrome characterized by fatty hyperaccumulation and steatosis of the liver without immoderate alcohol consumption. NAFLD is the commonest abnormal liver function disease at present, whose prevalence has doubled in the past 20 years, making it a health issue of global concern [1]. Furthermore, NAFLD involves a range of abnormal liver function diseases, ranging from the excessive accumulation of liver fat to steatohepatitis, with some cases eventually deteriorating into fibrosis and cirrhosis [2]. It is also closely linked to obesity and other metabolic syndromes, including hypertension, dyslipidemia and insulin resistance (IR).

As a severe challenge in the field of modern medicine, the pathogenesis of NAFLD is complicated and sustained through the joint regulation of a range of environmental, nutritional, metabolic, and gut microbiota factors [3]. The initial “two-hit hypothesis” suggests that the first hit mainly relates to the excessive accumulation of lipids in liver cells, while the second hit includes inflammation and oxidative stress [4]. Furthermore, recent studies have demonstrated that the gut microbiota is involved in the modulation of NAFLD and its related metabolic disorders [5]. The term “gut–liver axis” was coined to emphasize the close relationship between the gut and the liver. The gut microbiota can not only provide the host with energy via microbial metabolites, such as short-chain fatty acids (SCFAs), but also regulate the signaling pathways related to liver lipid metabolism [6]. Therefore, the treatment strategies for NAFLD mainly focus on regulating the gut microbiota to improve lipid disorder and hepatic steatosis, thereby further sustaining the normal function of the gut and liver.

Diet control, nutrition optimization, exercise and drug treatment are the main conventional treatments of NAFLD. At present, the most widely used lipid-lowering drugs mainly include statins and 3-hydroxy-3-methylglutaryl-CoA reductase inhibitors, which can lower the cardiovascular disease incidence rates and mortality in high-risk groups. However, statins can adversely affect muscle due to the inhibition of ubiquitin formation [7]. Therefore, it is urgent to develop a natural treatment for NAFLD that has superior preventive effects and a low risk of side effects. In the past few years, more and more attention has been paid to the extraction and identification of functional components from natural organisms, such as polyphenols and polysaccharides, as well as their extensive biological effects [8,9,10]. Among them, it is worth emphasizing that chondroitin sulfate (CS) has been identified as a novel dietary supplement and functional food, and it has become a recent focus of research [11].

CS is a sulfated glycosaminoglycan that is mainly obtained from the cartilage, bone and cornea of animals, and is the product of substituting sulfate groups at different positions in chondroitin [12]. CS has been widely studied for its antioxidant, antitumor, anti-inflammatory and anticoagulant effects [13]. Furthermore, extensive evidence has indicated that sulfated polysaccharides could regulate lipid metabolism and relieve metabolic diseases through multifunctional roles. Sulfated polysaccharides extracted from sea cucumbers can alleviate the metabolic syndrome caused by a high-fat diet (HFD) by lowering the levels of serum glucose, triglycerides (TG) and total cholesterol (TC), reducing body weight gain and inhibiting IR. In addition, studies have shown that the potency of CS depends on its type and molecular weight (MW) [14]. High-MW CS from skate cartilage mainly inhibits dietary fat absorption in the gut by inhibiting lipase activity, while low-MW CS is more easily absorbed and can inhibit lipid accumulation at lower doses [15]. Furthermore, the fermentation of CS and its derivative via the gut bacteria was proven to enhance the abundance of Lactobacillus and SCFA-producing bacteria and produce a significant amount of SCFAs, so as to alleviate metabolic syndrome [11].

Tilapia is a freshwater fish mainly cultured in the world aquaculture industry, and its processed products are mainly frozen tilapia fillets. In tilapia processing, many byproducts, such as fish heads, skin and bone are usually discarded and unused, which has caused enormous environmental problems. However, tilapia heads are rich in CS and may be developed and utilized as raw materials for aquatic health food [16]. Moreover, the effect and mechanism of tilapia-head chondroitin sulfate (TH-CS) on NAFLD are still unclear. Therefore, we extracted low MW TH-CS from tilapia heads, explored its role and elucidated the probable mechanisms from the perspective of the gut–liver axis in preventing hepatic and metabolic disorders in NAFLD mice. Thus, this study could provide theoretical guidance for the development of functional foods from aquatic animal processing byproducts to prevent hyperlipidemia.

## 2. Materials and Methods

### 2.1. Materials

Tilapias were purchased from Hainan Xiangtai Fishery Co., LTD (Hainan, China) and stored at −20 °C. High-fat feed was purchased from Hunan Slac Jingda Laboratory Animal Co., Ltd. (Changsha, China). Enzyme-linked immunosorbent assay kits for insulin (INS), adiponectin (ADPN), leptin (LEP), IL-6, tumor necrosis factor-alpha (TNF-*α*) and free fatty acids (FFAs) were acquired from Shanghai Enzyme-linked Biotechnology Inc. (Shanghai, China). Commercial kits for aminotransferase (ALT), aminotransferase (AST), TC, TG, HDL-C, LDL-C, glutathione (GSH), superoxide dismutase (SOD) and malonaldehyde (MDA) were purchased from Nanjing Jiancheng Bioengineering Institute (Nanjing, China). Antibodies against SREBP-1c were obtained from Santa Cruz Biotechnology Inc. (CA, USA). Antibodies against AMP-activated protein kinase (AMPK), phospho-AMPK, peroxisome proliferator-activated receptor (PPARγ) and fatty acid synthase (FAS) were obtained from Cell Signaling Technology (Beverly, MA, USA). Mouse anti-*β*-actin antibody and goat anti-mouse lgG-HRP secondary antibody were obtained from Abcam Inc. (Cambridge, UK). All other chemicals and reagents used were of analytical grade.

### 2.2. Extraction of TH-CS

The tilapia heads were homogenized after cleaning and sonicated with 95% (*v*/*v*) ethanol at the ratio of 1:10 (tilapia head: ethanol, *w*/*v*) for 1.5 h three times, with subsequent vacuum filtration. Next, the solid residues obtained from the filtration were added to 1% NaOH (*w*/*v*) at a ratio of 1:5. The mixture was incubated in a water bath at 50 °C for 7 h and intermittently stirred. After filtration, the filtrate was collected and the filter residue was treated again in the same way. The two filtrates were mixed and adjusted to pH 4 to remove acidic protein. Next, 0.1% (*w*/*v*) complex protease was added to the filtrate and kept at 50 °C for 3 h. The mixture was boiled for 10 min to inactivate the complex protease. Trichloroacetic acid was added to the mixture at a final concentration of 10% (*w*/*v*) to remove the protein. The precipitated protein was removed by centrifugation at 4000× *g* for 15 min at room temperature. The obtained supernatant was heated to 65 °C, and preheated H_2_O_2_ was added to react for 10 h, during which the pH was maintained at 5. Ethanol was added at a final concentration of 70% (*v*/*v*) to the mixture, incubated at 4 °C for 18 h to obtain the precipitate. The precipitate was collected by centrifugation at 6000× *g* for 10 min at room temperature. The collected precipitate was re-dissolved in distilled water to filter through a stirred ultrafiltration unit UFSC05001 (Merck Millipore, Darmstadt, Germany) to remove salt and other contaminants. The purified fractions were freeze-dried for further analysis. According to the method described by Kosakai et al. [17], the purity of TH-CS was determined by the carbazole reaction.

### 2.3. Characterization of TH-CS

#### 2.3.1. FT-IR Analysis

The FT-IR spectrum of TH-CS was determined using an FT-IR spectrometer (Bruker, Karlsruhe, Germany). The dried TH-CS freeze-dried powder was ground with potassium bromide powder at the ratio of 1:50 (TH-CS: potassium bromide, *w*/*w*) and then pressed to a transparent film. The film was scanned in the frequency range of 500–4000 cm^−1^ three times.

#### 2.3.2. TH-CS Molecular Weight Determination

The average MW of TH-CS was determined by the high-performance gel permeation chromatography (HPGPC) method by using a Shimadzu LC-10A instrument (Shimadzu, Kyoto, Japan) equipped with a BRT105-104-102 (8 mm× 300 mm) column. A total of 0.05 M NaCl was selected as the mobile phase, which flowed at a 0.6 mL/min rate. Dextrans with different MWs (5000, 11,600, 23,800, 48,600, 80,900, and 148,000 Da) were used as standards to obtain calibration curves by plotting the logarithm of retention time against their respective MW. Eventually, the MW of TH-CS was calculated according to the calibration curve.

#### 2.3.3. Monosaccharide Composition Determination

The sample was treated following the method described by Corrado et al. with a few modifications to analyse monosaccharide composition [18]. In brief, 10 mL of trifluoroacetic acid (3 M) was added to 10 mg TH-CS, mixed evenly, and hydrolyzed at 120 °C for 3 h. Next, the hydrolysate was dried under N_2_ flow and re-dissolved with 100 mL of ultrapure water. After the mixture was centrifuged at 10,000× *g* for 10 min, the obtained supernatant was analysed by an ICS5000 ion chromatograph (Thermo Fisher Scientific, Waltham, MA, USA) equipped with an electrochemical detector. The chromatographic conditions used in the experiment were as follows: column, DionexCarbopacTMPA20 (3 mm× 150 mm); mobile phase A: H_2_O; mobile phase B: 250 mM NaOH; mobile phase C: 50 mM NaOH and 500 mM sodium acetate; and flow rate, 0.3 mL/min. Standard monosaccharides, including mannose (Man), galactose (Gal), rhamnose (Rha), glucose (Glc), glucuronic acid (GlcA), arabinose (Ara), D-fructose (Fru), xylose (Xyl), fucose (Fuc) and glucosamine (GlcN) were analysed under the same conditions as a reference.

#### 2.3.4. Nuclear Magnetic Resonance (NMR) Spectroscopy

NMR spectroscopy was conducted on a Bruker AVANCE 600 MHz spectrometer to characterize the chemical structure of TH-CS. Prior to the NMR analysis, 20 mg of sample was dissolved in 0.6 mL D_2_O. The spectrometer frequency for NMR was 600 MHz.

### 2.4. Animals and Treatment

Male C57BL/6 mice (18.0 ± 2.0 g) were obtained from TianQin Biotechnology Company of Changsha with Licensed ID: SCXK2019-0014 (Changsha, China). The mice were kept in cages under conditions of 22 °C, 55% relative humidity, with a daily light/dark cycle of 12 h. Mice were adapted to these conditions for one week before the experiment.

Mice were distributed into four groups randomly (*n* = 8): the normal control group (“Control”), the HFD-induced NAFLD group (“Model”), the NAFLD simvastatin (ST) group (“ST”), and the NAFLD TH-CS group (“TH-CS”). The control group was fed a standard diet while the other three groups were fed a HFD. The composition of the standard diet was crude protein (18%), crude fat (4%), crude fiber (5%), coarse ash powder (8%), moisture content (10%), calcium (1.0–1.8%) and phosphorus (0.6–1.2%). HFD was composed of standard diet (78.8%), lard (10%), egg yolk powder (10%), cholesterol (1%) and sodium cholate (0.2%). The mice in the ST group were treated orally with ST at a dosage of 3 mg/kg/day, and those in the TH-CS group were treated orally with 80 mg/kg/day TH-CS. The experiment lasted for 8 weeks, and the weight and body length were recorded once a week.

At the end of the 8-week treatment, mice were euthanized under anesthesia using pentobarbital. Blood samples of mice were collected by eyeball extraction, and serum samples were separated by centrifugation at 2500× *g* for 15 min at 4 °C. The livers of mice were immediately dissected, weighed and examined grossly. The left lobe of liver samples was fixed in 4% paraformaldehyde for histopathological analysis, and the remaining liver tissue was stored at −80 °C for subsequent analysis after being flash-frozen in liquid nitrogen. Lee’s index and the liver index of mice were calculated via the following formulas.

(1) $$\begin{array}{c} {\;\mathrm{Lee}}^{’}s\;\mathrm{index}=\left[\mathrm{body}\;\mathrm{weight}\;{\left(g\right)}^{\frac{1}{3}}\;\times \;1000\right]/\mathrm{body}\;\mathrm{length}\;\left(\mathrm{cm}\right) \end{array}$$

(2) $$\begin{array}{c} \;\mathrm{Liver}\;\mathrm{index}=\mathrm{liver}\;\mathrm{weight}\;\left(g\right)/\mathrm{body}\;\mathrm{weight}\;\left(g\right)\;\times \;100\% \end{array}$$

### 2.5. Determination of IR Index

Fasting serum glucose was determined using commercially available blood glucose test strips and an ultrasensitive mouse insulin ELISA kit was used to determine fasting serum insulin in mice. The homeostasis model assessment index for insulin resistance (HOMA-IR) was calculated using Formula (3) to determine IR.

(3) $$\begin{array}{c} \;\mathrm{HOMA}-\mathrm{IR}\;\mathrm{index}=\left[\mathrm{fasting}\;\mathrm{glucose}\;\left(\mathrm{mM}\right)\;\times \;\mathrm{fasting}\;\mathrm{insulin}\;\left(\mu U/\mathrm{mL}\right)\right]/22.5 \end{array}$$

### 2.6. Serum and Hepatic Biochemical Assay

According to the instructions, commercially available kits were used to measure the expression of SOD, GSH, MDA, IL-6, TNF-*α* and FFA in the liver homogenate supernatant. The concentrations of serum TG, TC, HDL-C, LDL-C, ADPN, and LEP and enzyme activities of ALT and AST were also determined using commercial kits.

### 2.7. Haematoxylin and Eosin (H&E) Staining and Oil Red O Staining

H&E staining: the 4% paraformaldehyde was used for liver tissue fixation. The liver tissues were then implanted into paraffin wax blocks after being dehydrated by a series of alcohol concentrations. These blocks were divided into 5-micrometer sections rapidly and stained with H&E for histological analysis. For oil red O staining, the liver tissues were fixed in 4% paraformaldehyde and dehydrated in sucrose solution. Next, they were embedded in the composite material and sliced. After staining with oil red, they were viewed at 400× magnification through an Olympus microscope (Olympus, Beijing, China).

### 2.8. Gut Microbiota Analysis

A 16S-rDNA gene analysis was used to identify the gut microbiota diversity and composition of each group, which were collected from caecal content samples. Briefly, microbial DNA was extracted and checked by 1% agarose gel electrophoresis to evaluate its concentration and integrity, and after 16S amplification of the V3–V4 hypervariable region. The PCR products were sequenced on an Illumina HiSeq2500 platform to analyze the structure of gut microbiota after being quantified and purified. The chao1 and ACE were used to assess the *α*-diversity among groups. To compare *β*-diversity, nonmetric multidimensional scaling (NMDS) was performed using OTUs for each sample based on Bray–Curtis. The 16S rDNA of caecal content samples were submitted to the NCBI database (SRA accession: PRJNA787458).

### 2.9. Quantification of SCFAs

Gas chromatography was used to quantitatively analyse the concentrations of SCFAs, which were collected from caecal content samples. The samples were homogenized in ultrapure water at a ratio of 1:10 (caecal content: ultrapure water, *w*/*v*) and centrifuged at 13,000× *g* for 20 min at 4 °C. The internal standard was set by the mixture of filtered supernatant and 2-ethy acid. Next, the filtrate was injected into an Agilent 8890b-5977b gas chromatography-mass spectrometer (Agilent Technologies, Santa Clara, CA, USA) equipped with a flame ionization detector and an HP-FFAP column (30 m × 0.25 mm × 0.25 μm; Agilent) capillary column. The setting temperature of the injector and detector was 260 °C. The oven temperature was initially set at 80 °C, then raised to 120 °C at a rate of 40 °C/min, raised to 230 °C at a rate of 10 °C/min, and finally held at 230 °C for 3 min. The column flow rate of He which worked as the carrier gas was 1 mL/min and the split ratio was 10:1. A standard curve was constructed with different concentrations of a standard mix containing acetate, propionate, butyric acid, isobutyrate, pentanoic acid and isovaleric acid.

### 2.10. Quantitative RT-PCR

Total RNA from mouse liver tissue was extracted by an Eastep Super Total RNA Extraction kit (Promega, Shanghai, China). Next, single-stranded cDNA was generated from 1 μg total RNA by reverse transcription by using a RevertAid First Strand cDNA Synthesis kit (Thermo Fisher Scientific). A SuperReal PreMix Plus (SYBR Green) (TIANGEN Biotech) was used for quantitative PCR amplification. PCR was performed in a 20-microliter system. The gene-specific primers used are shown in Table 1. Eventually, the data obtained were normalized, analysed and compared with the internal reference gene GAPDH.

### 2.11. Western Blotting

The concentration of isolated total protein of liver tissue homogenate was measured by BCA assay (Beyotime, Beijing, China). Subsequently, tissue lysates with equal protein amounts were separated by SDS-polyacrylamide gel electrophoresis (EpiZyme, Shanghai, China) and blotted to PVDF membranes, which were incubated for 12 h with different specific primary antibodies after blocking with 5% nonfat milk solution. The membranes were then incubated with a peroxidase-labeled secondary antibody, and an ultrasensitive ECL Chemiluminescence Kit (Biosharp, Anhui, China) was used. Western blot bands were visualized using a Tanon-5200 multifunctional imaging system (Shanghai, China) and the relative density of the bands was quantified by optical density using Quantity One.

### 2.12. Statistical Analysis

Data are expressed as the mean ± SEM. Analysis of variance (ANOVA) was used to identify the significant differences between groups. For all analyses, the critical *p*-value was set to 0.05 and 0.01 for significant or extremely significant differences.

## 3. Results

### 3.1. Chemical Properties of TH-CS

**Figure 1:**
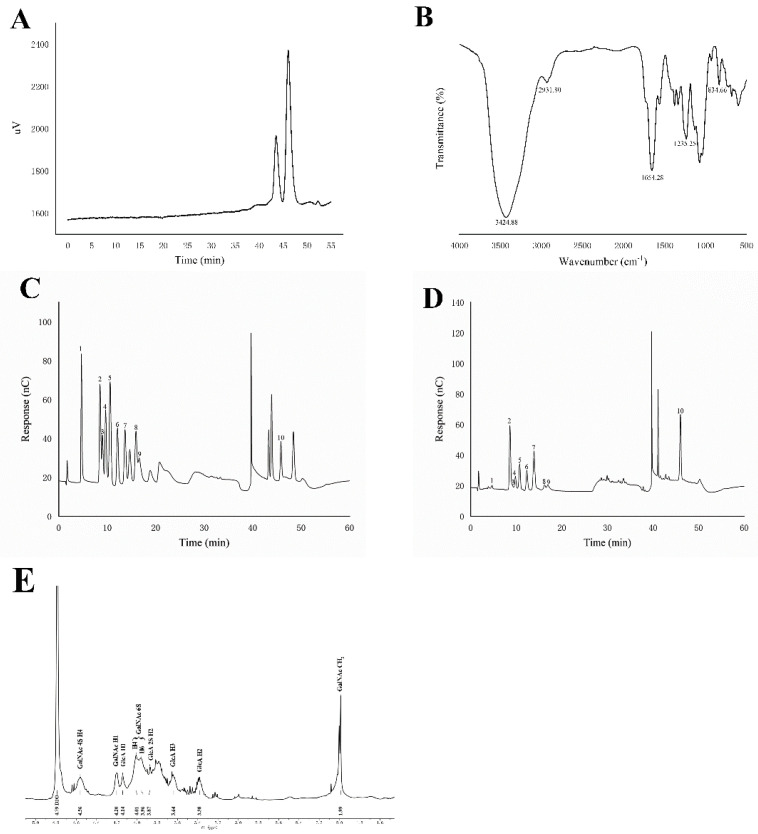
Chemical properties of TH-CS. (**A**) MW of TH-CS; (**B**) FT-IR analysis of TH-CS; (**C**) ion chromatograms of a mixture of 10 monosaccharide standards; (**D**) ion chromatograms of TH-CS. Peaks: (1) Fuc, (2) GalN, (3) Rha, (4) Ara, (5) GlcN, (6) Gal, (7) Glc, (8) Xyl, (9) Man and (10) GlcA; (**E**) ^1^H NMR spectra of TH-CS.

After calculation, the TH-CS yield in the present study was 0.44 ± 0.08% and the purity was 78.32 ± 0.14% (data not shown). Numerous studies have shown that the MW of polysaccharides is closely related to their biological activity. Specifically, polysaccharides with high MW generally have very poor water solubility, which makes it difficult to enter the organism to exert pharmacological effects and hinder their bioactivity [19]. The HPGPC showed that the average MW of the TH-CS was 6.2 kDa (Figure 1A), which is lower than that of other previously reported extracted polysaccharides. Thus, this CS shows excellent potential.

The FT-IR spectrum of the TH-CS is shown in Figure 1B. The strong absorption peak at approximately 3424.88 cm^−1^ could be ascribed to the O-H stretching vibrations, while the peak at 2931.80 cm^−1^ was attributed to the C-H stretching vibrations of -CH_2_ groups in the polysaccharide [20]. The strong absorption peak at 1654.28 cm^−1^ may have been caused by the C=O stretching vibration in the ester carbonyl [21]. The significant absorption appearing at 1262 cm^−1^ was attributed to the S=O asymmetric stretching vibration, revealing the presence of sulfates in the TH-CS [22]. The C-O-S stretching vibration band at 834.66 cm^−1^ indicated that the sulfate groups of TH-CS were bound to N-acetyl-galactosamine (GalNAc) in the C6 position, which illustrates the presence of chondroitin-6-sulfate (CS-C) in TH-CS [20].

As shown in Figure 1C,D, a compositional monosaccharide analysis showed that the TH-CS was composed of GlcA (43.1%), Glc (18.6%), Gal (8.9%), GalN (8.8%), GlcN (5.1%), Man (5.0%), Ara (4.8%), Xyl (3.0%), Rha (2.4%) and Fuc (0.3%). These results reveal that TH-CS is an acidic polysaccharide with GlcA, Glc and Gal as its major components.

NMR is a fast method that provides an overview of TH-CS composition (Figure 1E). Typical CS ^1^H NMR signals are concentrated in the region from 1.5 to 5.0 ppm. The signal at 1.99 ppm indicated the methyl group of the GalNAc structure [23]. Furthermore, the TH-CS signals at 4.14, 3.38 and 3.64 ppm were assigned to GlcA H1-H3, respectively. In addition, the spectra showed a predominance of the signals corresponding to a sulfation of 6 in the GalNAc (singlets of H4 and H6 at 4.01 and 3.96 ppm) as compared to a sulfation of 4 [24]. However, additional signals appeared from 2.2 to 3.3 ppm; this was most likely due to protein impurities. These signals were, however, of low intensity, suggesting that the purity of the TH-CS was at a relatively high level.

### 3.2. TH-CS Prevented Obesity in NAFLD Mice

**Figure 2:**
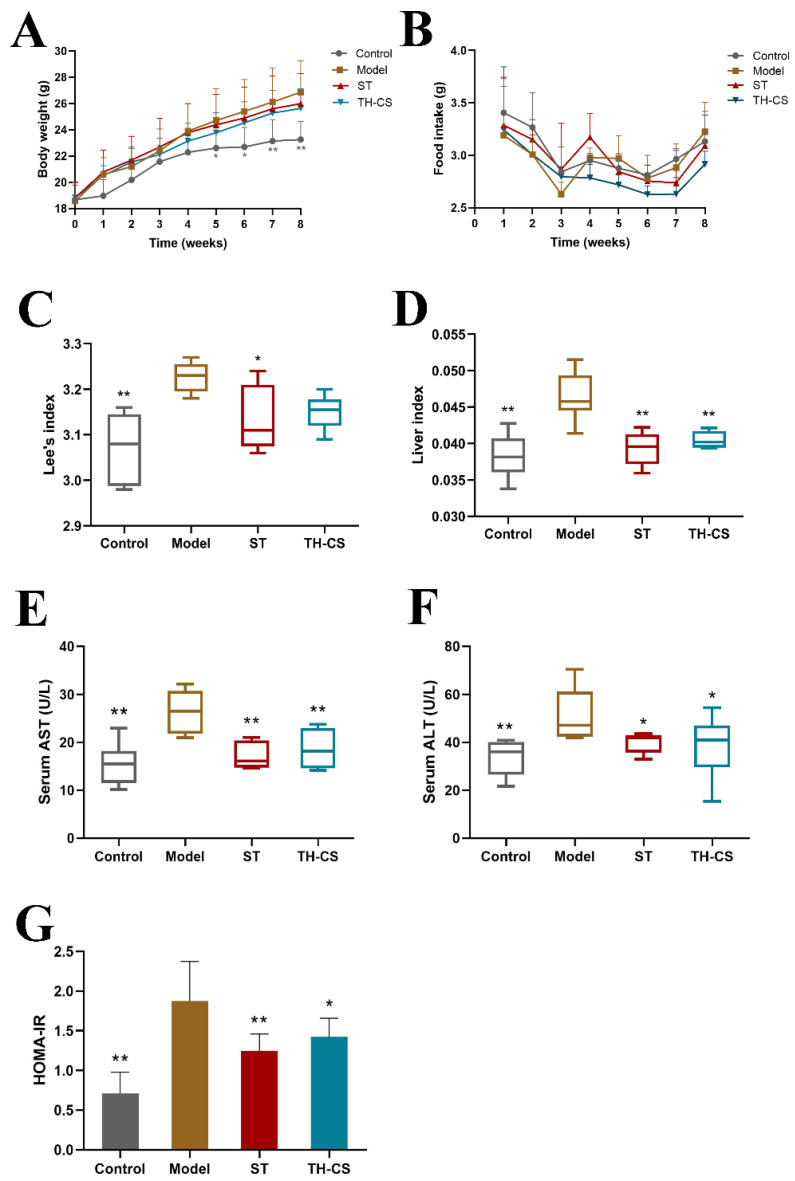
TH-CS alleviated obesity, liver function and IR in NAFLD mice. (**A**) Body weight; (**B**) food intake; (**C**) Lee’s index; (**D**) the liver index; (**E**) the levels of AST in the serum; (**F**) the levels of ALT in the serum; (**G**). HOMA-IR. Values were expressed as mean ± SEM in each group. * *p* < 0.05 as compared to the model group; ** *p* < 0.01 as compared to the model group. (*n* = 6).

In this study, the body weight, food intake, liver weight and body length of the mice were measured. As depicted in Figure 2A, during the 8 weeks of continuous administration, the body weights of the mice in all four groups increased gradually with time. However, the average rate of body weight gain of the Model group was higher than that of the other three groups. As a result, the average body weight of the mice treated with TH-CS was lower than that in the model group, beginning at the third week. The food intake did not vary remarkably among the control, model, ST and TH-CS groups (Figure 2B).

At the end of the experiment, Lee’s index and liver index values were dramatically higher in the model group than in the control group (Figure 2C,D). By contrast, the liver index value was markedly lower in the TH-CS group than in the model group, which indicated that the administration of TH-CS effectively suppressed the HFD-induced increase in liver weight. The Lee’s index value was slightly lower in the mice administered TH-CS than in the mice in the model group; however, the difference was not significant.

### 3.3. TH-CS Alleviated Liver Function in NAFLD Mice

The levels of AST and ALT are widely regarded as the “gold standard” indication of liver function [25]. Intracellular enzymes are released into the bloodstream and lead to an increase in serum levels, which is the most significant phenomenon in liver damage. Consequently, high values of serum ALT and AST may reflect liver damage. As shown in Figure 2E,F, the mice in the model group had a 70.9% and 52.9% increase in serum AST and ALT activities, respectively, indicating serious liver injury in the HFD mice. However, the TH-CS significantly reduced the increase in serum AST and ALT activities. It is worth mentioning that the TH-CS and ST were equivalent at reducing the activity of serum AST and ALT enzymes.

### 3.4. TH-CS Alleviated IR in NAFLD Mice

IR is at the core of the pathogenesis of metabolic syndrome. Therefore, controlling IR is the first but most crucial step in the treatment of NAFLD [26]. HOMA-IR is an index used to evaluate the level of IR. HFD significantly increased the HOMA-IR value of the model group (Figure 2G), indicating that the model group had severe IR. Furthermore, the HOMA-IR value of mice treated with TH-CS was substantially reduced by 24.41% compared to the model group, indicating the inhibition of IR.

### 3.5. TH-CS Attenuated Lipid Accumulation in the Sera and Livers of NAFLD Mice

**Figure 3:**
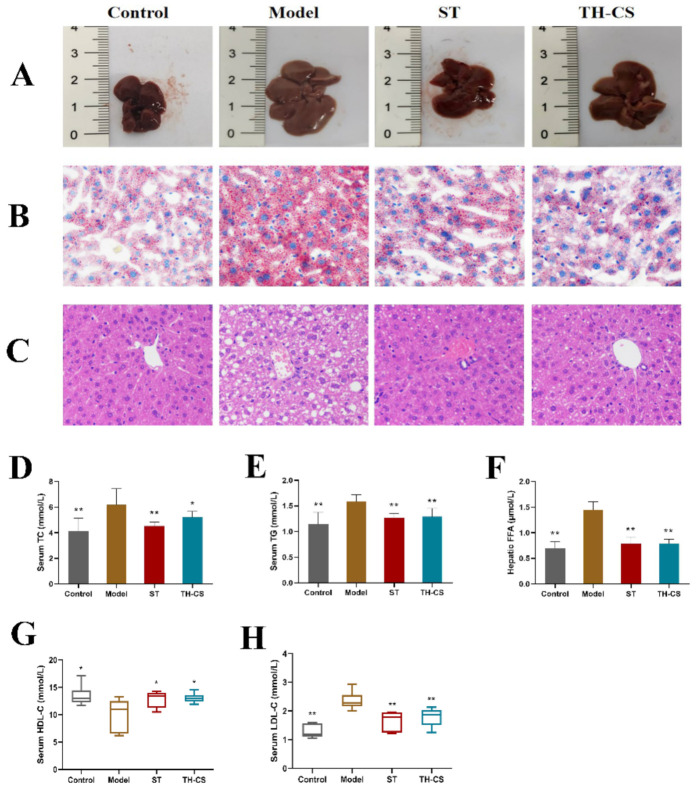
TH-CS attenuated lipid accumulation in sera and livers of NAFLD mice. (**A**) Changes in liver morphology; (**B**) oil red O staining of the liver histology was photographed at 400× magnification; (**C**) H&E staining of the liver histology was photographed at 400× magnification; (**D**) serum TC; (**E**) serum TG; (**F**) FFA level in liver; (**G**) serum HDL-C; (**H**) serum LDL-C. Values were expressed as mean ± SEM in each group. * *p* < 0.05 as compared to the model group; ** *p* < 0.01 as compared to the model group. (*n* = 6).

We evaluated whether the administration of TH-CS ameliorated the abnormal lipid metabolism in mice caused by HFD consumption. TG and TC are mainly synthesized in the liver, and the long-term intake of HFD results in TG and TC synthesis and secretion disorders. This imbalance of lipid metabolism leads to an increase in serum TG, TC and LDL-C levels and a decrease in HDL-C levels. As shown in Figure 3, we observed that the levels of TC, TG and LDL-C in the sera of NAFLD mice were significantly increased by 49.99%, 37.49% and 83.03% after 8 weeks of feeding with HFD, respectively, and the serum HDL-C concentrations were reduced by 20.17%. These results indicated that the lipid metabolism homeostasis in the mice was disturbed by the consumption of HFD for 8 weeks. As expected, both TH-CS and ST could significantly prevent these disturbances. Specifically, the administration of TH-CS inhibited the increases in TC, TG and LDL-C levels in serum. The contents of serum TC, TG and LDL-C were reduced by 15.94%, 18.48% and 23.19%, respectively, when compared with the model group.

FFAs seem to be influential mediators of lipotoxicity and potential cellular toxins, which can prompt excessive fat accumulation through IR. In addition, FFA stimulates the expression of TNF-*α* through the lysosomal pathway to promote hepatic lipotoxicity [27]. As reflected in Figure 3F, hepatic FFA levels showed a sharp rise in the model group when compared with the normal mice. However, the FFA level was decreased after TH-CS treatment.

As shown in Figure 3A, according to visual observations, the livers of the control group were normal in size and shape, and the colour was dark red. However, the liver of the model group was much larger and the surface colour was grey–yellow. After 8 weeks of administration, the livers of the ST group and TH-CS mice improved to a certain extent.

Liver histology was obtained by staining with H&E and oil red O. The mice in the model group developed pronounced steatosis and vacuolization when compared with control group in H&E sections (Figure 3B). The morphological structures of hepatocytes and macrovesicular steatosis of the liver in the NAFLD mice were significantly recovered by treatment with ST and TH-CS. The results of the oil red O staining indicated that TH-CS and ST treatment reduced the number of hepatic lipid droplets in the NAFLD mice, which further confirmed that TH-CS and ST could improve abnormal lipid accumulation (Figure 3C). Our results indicated that both TH-CS and ST treatment could ameliorate the hepatic steatosis caused by overnutrition to a certain extent.

### 3.6. TH-CS Reduced Liver Inflammation in NAFLD Mice

**Figure 4:**
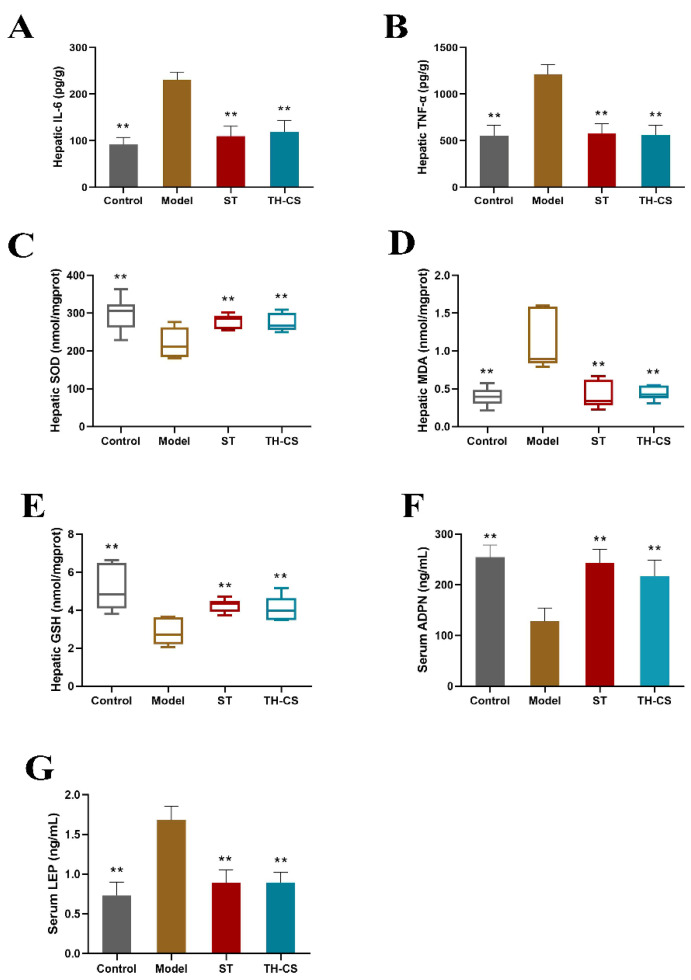
TH-CS regulates liver inflammation, hepatic oxidative stress and obesity factors in NAFLD mice. (**A**) Liver IL-6 level; (**B**) liver TNF-*α* level; (**C**) liver SOD level; (**D**) liver MDA level; (**E**) liver GSH level; (**F**) serum ADPN level; (**G**) serum LEP level. Values were expressed as mean ± SEM in each group. ** *p* < 0.01 as compared to the model group. (*n* = 6).

The deterioration of NAFLD is closely related to the development of inflammation. Downstream signals of Toll-like receptors, such as the NF-*κ*B signaling pathway, are activated by the influx of excess nutrients into the metabolic pathway and trigger the classic inflammatory pathway. The activation of the inflammatory pathway promotes the increase of proinflammatory cytokines, such as IL-6, IL-1, and TNF-*α* [28]. After 8 weeks, the hepatic levels of TNF-*α* and IL-6 were significantly higher in mice in the model group than in mice in the control group (Figure 4A,B). Both the ST and TH-CS groups had significantly lower hepatic TNF-*α* and IL-6 levels.

### 3.7. TH-CS Regulated Hepatic Oxidative Stress in NAFLD Mice

The levels of hepatic SOD, MDA and GSH were measured to investigate the role of oxidative stress in NAFLD (Figure 4C–E). As the main product of lipid peroxidation degradation, the level of MDA reflects the severity of tissue lipid peroxidation damage. SOD can prevent the damage caused by lipid peroxidation by scavenging free radicals in the body. Recognized as a vital indicator of the body’s antioxidant capacity, GSH can scavenge free radicals and is an essential nonenzymatic antioxidant in the body. The activity of SOD and GSH in NAFLD mice was decreased strongly when compared with the control group. By contrast, the TH-CS significantly inhibited the decrease in SOD and GSH activity caused by the HFD and exhibited a protective effect against lipid peroxidation. Moreover, the long-term consumption of HFD increased the level of MDA, but this increase was greatly inhibited by TH-CS treatment. These results indicate that TH-CS can effectively prevent oxidative stress in the livers of NAFLD mice.

### 3.8. TH-CS Affected Obesity Factors in NAFLD Mice

The incidence of NAFLD is intimately associated with obesity, excessive lipid accumulation and IR. As an endocrine organ, adipose tissue secretes cytokines, such as LEP and ADPN, which are essential in the progression of NAFLD. The mean levels of serum ADPN and LEP for the four groups of mice are shown in Figure 4F,G. At the end of the 8-week treatment, the levels of serum ADPN were markedly lower in the mice in the model group (127.94 ± 26.19 ng/mL) than in the mice in the control group (254.54 ± 23.94 ng/mL). The levels of serum LEP in the NAFLD mice were markedly increased, by 130.13%, compared with those in the normal mice. However, treatment with TH-CS markedly increased the serum ADPN levels in the mice in the TH-CS group. Meanwhile, the TH-CS reduced LEP secretion in the NAFLD mice. These results indicate the regulatory effect of TH-CS on obesity factors.

### 3.9. TH-CS Improved Gut Microbiota Composition in NAFLD Mice

**Figure 5:**
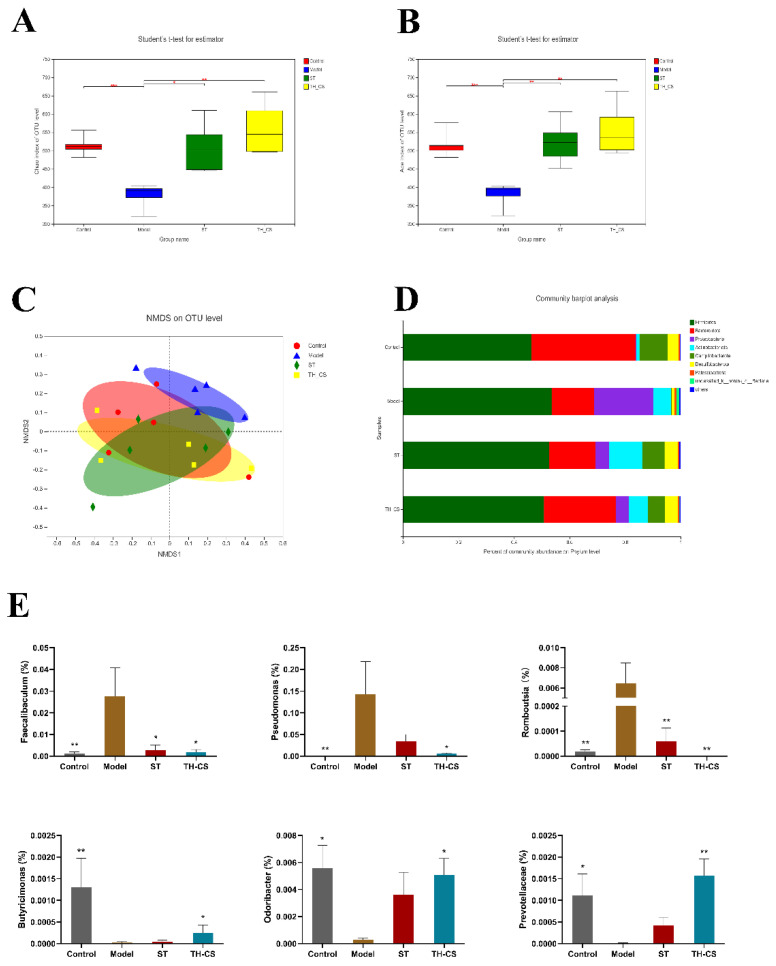
TH-CS moderates intestinal microecology in NAFLD mice. (**A**) Chao1 index; (**B**) ACE index; (**C**) nonmetric multidimensional scaling (NMDS) analysis based on Bray–Curtis distance; (**D**) microbial community profiling in the phylum level for each group; (**E**) the relative abundance of certain bacteria in genus level. Values were expressed as mean ± SEM in each group. * *p* < 0.05 as compared to the model group; ** *p* < 0.01 as compared to the model group. (*n* = 5).

As an essential factor in the development of and recovery from NAFLD, the gut microbiota recent area of significant research focus. Therefore, we explored the impact of TH-CS on the microbiota’s diversity and composition. To evaluate the alterations in the diversity of the bacterial species among the four groups, the *α*-diversity was measured with the Chao1 (Figure 5A) and ACE (Figure 5B) indices. Compared with the normal mice, the phylotype richness of the NAFLD mice’s gut microbiota was greatly decreased, based on the reduced Chao1 and ACE values. Nevertheless, the phylotype richness was significantly restored by treatment with TH-CS, indicating that oral TH-CS could effectively prevent the reduction in intestinal bacterial species caused by HFD. Furthermore, the *β*-diversity was used to analyse the overall structural changes in the gut microbiota. The results of the NMDS-based *β*-diversity analysis indicated that the overall structure of the gut microbiota of the NAFLD mice was significantly changed, while the TH-CS-administered group clustered separately from the model group and shifted toward the control group (Figure 5C). These results suggested that TH-CS could restore the homeostasis of the gut microbiota in NAFLD mice induced by HFD and showed the ability to regulate the structure of the gut microbiota.

In the meantime, further analysis was performed to reveal the detailed differences in the relative abundance of bacterial communities at the phylum and genus levels. Figure 5D mainly shows the eight most abundant phylums in the caecum content of the mice; *Bacteroidetes*, *Firmicutes* and *Proteobacteria* accounted for the largest proportion. The relative abundance of the gut microbiota in the NAFLD mice was significantly different from that in the normal mice. The abundance of *Bacteroides* rose, while those of *Firmicutes* and *Proteobacteria* decreased to varying degrees in the ST and TH-CS groups when compared with the model group. Subsequently, we identified distinctions between the four groups at the genus level. By comparison with model group, the ratio of the genera *Faecalibaculum*, *Pseudomonas* and *Romboutsia* decreased substantially, while the relative abundance of SCFA-producing bacteria, such as *Butyricimonas*, *Odoribacter* and *Prevotellaceae*, was enhanced in the TH-CS group (Figure 5E). Moreover, the TH-CS showed a better effect than ST on the ratio of SCFA-producing bacteria. The above results indicated that supplementation with TH-CS could regulate the structure of the gut microbiota, selectively promote the proliferation of probiotics (especially SCFA-producing bacteria) and improve the imbalance of gut microbiota induced by HFD.

### 3.10. TH-CS Regulated the Levels of SCFAs in NAFLD Mice

**Figure 6:**
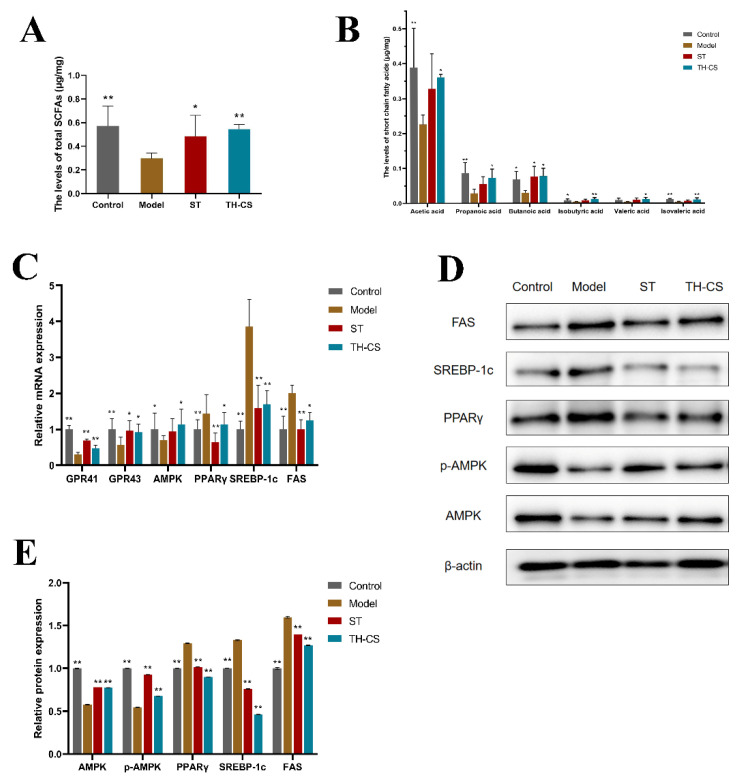
TH-CS modulates the levels of SCFAs and metabolic gene expression in NAFLD mice. (**A**) The total levels of SCFAs; (**B**) the levels of specific SCFAs; (**C**) the mRNA expressions of GPR41, GPR43, AMPK, PPAR*γ*, SREBP-1c and FAS. (**D**) Representative images of the Western blotting for AMPK, p-AMPK, PPARγ, SREBP-1c, FAS in the liver, with *β*-actin applied as a loading control. (**E**) The protein expressions of AMPK, phosphorylation of AMPK, SREBP-1c, PPAR*γ* and FAS. Values were expressed as mean ± SEM in each group. * *p* < 0.05 as compared to the model group; ** *p* < 0.01 as compared to the model group. (*n* = 6).

SCFAs are final products of the indigestible foods fermented by intestinal microbes and play a role in regulating balance, inflammatory processes and obesity. Therefore, the types and contents of SCFAs of the mice’s caecal contents were measured at the end of the experiment. The total SCFA content in the model group was reduced when compared with that of the normal mice, but it was significantly higher in the TH-CS group and ST group than in the model group (Figure 6A). Specifically, TH-CS treatment markedly increased the contents of acetic acid, propanoic acid, butanoic acid, isobutyric acid, valeric acid and isovaleric acid in the NAFLD mice (Figure 6B). However, ST treatment did not markedly enhance the amounts of the SCFAs, except for butyric acid, in the caecal contents of the NAFLD mice. In short, these data indicate that TH-CS could stimulate the production of SCFAs and that its effect was significantly better than that of ST.

### 3.11. TH-CS Inhibited Hepatic Lipid Accumulation via Modulation of Metabolic Gene Expression in NAFLD Mice

To investigate the underlying molecular mechanism of lipid metabolism modulated by TH-CS, the expression of SCFA receptors and hepatic lipid metabolism genes were detected in the liver via qPCR and WB methods (Figure 6C–E).

GPR41 and GPR43 are a couple of G protein-coupled receptors activated by SCFAs that are related to lipid synthesis. The expression levels of GPR41 and GPR43 were increased significantly in the livers of the TH-CS-treated mice compared with the NAFLD mice.

Because the AMPK-SREBP1 pathway plays an essential role in lipid metabolism, we explored whether the alleviating effect of TH-CS on NAFLD was associated with the expression of AMPK, the phosphorylation of AMPK, SREBP-1c and its downstream target genes FAS in the HFD-fed mice. AMPK is not only a cellular sensor that is indispensable to the restoration of cellular energy homeostasis; it is also a central regulator of multiple metabolic pathways. Therefore, it is considered as a therapeutic target for NAFLD [29]. The expression of AMPK was significantly decreased in the NAFLD mice, while it was significantly increased in the TH-CS treated mice. Meanwhile, the mRNA expression of SREBP-1c and FAS was noticeably upregulated in the mice fed with HFD. However, TH-CS treatment significantly lowered the mRNA levels of these molecules. Furthermore, the liver protein levels of AMPK, SREBP-1c and FAS were nearly consistent with the mRNA levels. PPAR*γ* is closely associated with obesity, adipocyte differentiation and IR. Our research showed that the expression of PPAR*γ* in the livers of the NAFLD mice was significantly increased, whereas the expression of PPAR*γ* in the TH-CS group was noticeably decreased.

## 4. Discussion

With the prevalence of sedentary lifestyles and HFD, the incidence of NAFLD increases, making it a significant health concern. Thus, it is urgent to develop specific therapeutic drugs for NAFLD to reverse the occurrence and development of NAFLD. In recent years, various polysaccharides have been proven to be useful in fatty liver, hyperlipidemia and hyperglycemia [30]. CS is rich in sulfate, which shows stronger biological activity than other polysaccharides in preventing and treating NAFLD. CS can regulate lipid metabolism and it can be degraded by the gut microbiota to produce metabolites, such as SCFAs and free sulfate, which affect the composition of gut microbiota in turn. By contrast, the CS from aquatic products is safer due to the presence of diseases such as mad cow disease, pig cholera and foot-and-mouth disease in terrestrial animals. However, commercial CS is mainly extracted from sharks, whose resources are rare and expensive. Therefore, looking for a new source of chondroitin sulfate with high economy and safety has attracted extensive attention from scholars. Here, we extracted TH-CS with low MW (MW = 6.2 kDa), which is an acidic polysaccharide mainly composed of GlcA, Glc, Gal and GalN from tilapia heads, in molar ratios of 4.89:2.11:1.01:1.00. Moreover, the preventive and restorative activities of TH-CS against NAFLD were demonstrated in this study.

The pathogenesis of NAFLD is generally considered to include a two-hit model. Abnormal hepatocellular lipid accumulation and IR are the first “hit” [31]. With an excessive intake of fatty acids, HFD can lead to obesity, NAFLD, and other metabolic syndromes. In this research, by using a NAFLD model induced with HFD, we found that the model mice had significant weight gain, intrahepatic lipid accumulation and dyslipidemia. These symptoms were consistent with a previous study that identified metabolic syndrome [32]. As expected, TH-CS significantly reduced the liver index values in the HFD mice. In addition, TH-CS improved the serum lipid levels and distinctly lowered liver fat accumulation in the NAFLD mice, particularly by lowering the levels of TG, TC and LDL-C. TH-CS treatment also reduced NAFLD features, as it lowered the levels of ALT and AST to reduce the liver damage in the NAFLD mice. Furthermore, the results of histological staining in the livers further indicated that the TH-CS significantly improved lipid accumulation in the liver. These data indicate that TH-CS could effectively inhibit and improve the first “hit” of NAFLD.

Due to the causal relationship between liver fat accumulation and IR, IR has become a key factor in the first “hit” of NAFLD. When IR occurs, the insulin regulation of fat metabolism is weakened, resulting in an increase in FFAs released by adipose tissue decomposition [33]. Increased FFAs cause lipid peroxidation damage by activating mitochondrial unsaturated fatty acid oxidation, leading to fatty liver formation. Additionally, this can aggravate IR by inhibiting insulin signal transduction and reducing insulin clearance, forming a vicious cycle and, ultimately, influencing lipid metabolism and inducing NAFLD progression [34]. Thus, studying the potential action of TH-CS on IR regulation is of profound significance. In the present study, we discovered that HFD resulted in a significant increase in hepatic FFA levels in mice, whereas TH-CS treatment effectively reduced hepatic FFA levels. Moreover, treatment with TH-CS greatly enhanced insulin sensitivity compared to the model group, which was in line with previously reported results [35].

The “second hit” of NAFLD is considered to be linked to inflammation and oxidative stress. Based on the accumulation of TGs, inflammation and oxidative stress reactions occur in the liver, leading to hepatocyte degeneration and necrosis. According to a previous study, the intake of sulfated polysaccharides derived from brown seaweeds decreased the hepatic mRNA expression of IL-1*β* and TNF-*α* in NAFLD rats [28]. In agreement with these findings, the TH-CS treatment significantly reduced the levels of TNF-*α* and IL-6 in the NAFLD mice, suggesting that the TH-CS effectively protected liver cells from the inflammatory effects caused by HFD intake. Moreover, according to one report, CS derived from skate cartilage ameliorated hyperlipidaemia-induced oxidative stress [36]. Similarly, TH-CS inhibited the formation of MDA and ameliorated the levels of SOD and GSH in the livers of the NAFLD mice, indicating that the TH-CS inhibited oxidative stress and lipid peroxidation.

Many studies have demonstrated that the development of NAFLD is directly related to imbalances in the gut microbiota. Wu et al. prepared abalone sulfated polysaccharide and its desulfurization products and analyzed the contribution of sulfate groups through a mouse model. They found that the presence of sulfate groups could lead to some differences in the gut microbiota and its metabolites, thus forming differences in biological activity with other polysaccharides [37]. In the current study, from the results of the *α*- and *β*-diversity analyses, it could be seen that TH-CS significantly reshaped the gut microbiota structure and enhanced the microbial diversity of the NAFLD mice. In addition, the TH-CS significantly modified the composition of the gut microbiota at different levels of classification. In particular, TH-CS supplementation could significantly enhance the relative abundance of SCFA-producing bacteria, such as *Butyricimonas*, *Odoribacter* and *Prevotellaceae*. It was previously reported that polysaccharides cannot be degraded in the gastrointestinal tract after oral administration, but that they can be fermented by the gut microbiota and generate SCFAs to regulate inflammation and lipid metabolism, which is a vital mechanism for indigestible polysaccharides in the prevention of HFD-induced NAFLD [38]. In the current study, the results of targeted metabonomics for SCFAs further revealed the manipulation effect of TH-CS treatment on the gut microbiota. The results demonstrated that the SCFA contents, especially those f propionic acid and butyric acid, in the mouse caecum were improved by TH-CS treatment. Its effect was significantly better than that of ST treatment, showing the great potential of natural aquatic compounds to improve NAFLD. Correspondingly, the mRNA expression of GPR41 and GPR43 in the livers of the NAFLD mice increased significantly after 8 weeks of treatment with TH-CS. GPR41 and GPR43 are a pair of G protein-coupled receptors, which can be activated by SCFAs, such as acetic acid, propionic acid and butyric acid. They have been shown to be related to gut-microbial-mediated connections in a variety of diseases, such as liver steatosis, diabetes and inflammation. This unique ligand specificity suggests that SCFAs may mediate the interaction between the gut and the liver. In short, TH-CS may enhance the contents of SCFAs by promoting the bacterial production of SCFAs in the intestines, thereby greatly contributing to the prevention of NAFLD.

It is widely known that the gut microbiota and its metabolites, such as SCFAs, play a vital role in regulating lipid metabolism. In particular, SCFAs can offer energy to the host and regulate the expression of lipid-metabolism-related proteins as signal molecules [39]. Several studies have shown that AMPK, a key enzyme in energy metabolism, can be mediated by SCFAs and plays a crucial role in regulating lipid metabolism by sensitizing its phosphorylation activity [40]. Phosphorylated AMPK can effectively inhibit the production of key lipid synthesis enzymes and hinder the expression of the lipid-synthesis genes SREBP-1c and FAS. These genes are downstream target genes of AMPK and make a valuable contribution to the regulation of TC, TG and FFA synthesis in the liver. In agreement with this, Li et al. reported that fucosylated CS from sea cucumbers could balance lipid transport by normalizing the expression of genes involved in the lipid metabolism pathway [41]. In this study, the TH-CS greatly improved the protein expression of phosphorylated AMPK and reduced the protein expression of SREBP-1c and FAS. PPAR*γ* is another key transcription factor; it is a nuclear receptor that can regulate lipid metabolism disorder by modulating free fatty acids. At the same time, PPAR*γ* is related to the regulation of obesity factors, such as ADPN and LEP; changes in obesity factors are the best predictors of insulin sensitivity [42]. In the present study, the mice treated with TH-CS had a remarkable increase in serum ADPN levels and decreases in serum LEP concentrations and expression of PPAR*γ* in their liver tissue. The above results suggest that TH-CS can adjust the secretion of FFAs and obesity factors by decreasing the mRNA and protein levels of PPAR*γ*, thereby improving IR.

## 5. Conclusions

In this study, we extracted TH-CS from tilapia heads and explored its lipid-lowering effects and mechanisms in a HFD-induced NAFLD mouse model. The TH-CS exerted a protective effect against IR and lipid metabolism disorder. At the same time, the TH-CS treatment significantly reduced inflammatory responses in the liver and oxidative stress. These effects appear to have been mediated by the regulation of the gut–liver axis homeostasis modulation in the NAFLD mice, i.e., the gut microbiota, microbial metabolites and related lipid metabolism signaling pathways. TH-CS could modulate the structure and composition of the gut microbiota as well as stimulating related bacteria to produce SCFAs. Moreover, after degradation to SCFAs by the gut microbiota, the TH-CS regulated the AMPK-SREBP1 signaling pathway and the level of PPARγ in the liver to alleviate lipid accumulation and IR in the NAFLD mice. In short, we demonstrated that TH-CS can effectively alleviate HFD-induced NAFLD in mice. Our findings support the comprehensive utilization of the byproducts of aquatic animal processing and provide a theoretical basis for the use of TH-CS as a nutritional supplement for ameliorating NAFLD.

## Figures and Tables

**Table 1:** Primer sequences of Real-time PCR.

Gene	Forward Primer	Reverse Primer
GPR41	ACCACTATTTACCTCACCTCCCTCTTC	CGATGCCAGGAACCAACAGACTAC
GPR43	TGCACCATCGTCATCATCGTTCAG	AGTTCTCGTAGCAGGTTATTTGGTTCTC
AMPK	TCCACAGAGATCGGGATCAGTTAGC	GAGTTAGGTCAACAGGAGAAGAGTCAAG
PPAR*γ*	AGCCCTTTACCACAGTTGATTTCTCC	GCAGGTTCTACTTTGATCGCACTTTG
SREBP-1c	CCTGCTTGGCTCTTCTCTTTGTCTAC	AGGTCAGCTTGTTTGCGATGTCTC
FAS	GTTTAAAGCTGAGGAGGCGGGTTC	GTTTTCAGGTTGGCATGGTTGACAG
GAPDH	AAGAAGGTGGTGAAGCAGGCATC	CGGCATCGAAGGTGGAAGAGTG

## Data Availability

All raw data supporting reported results is available from authors upon request.
